# Insomnia in the third trimester and its association with postpartum anxiety

**DOI:** 10.1192/j.eurpsy.2025.1036

**Published:** 2025-08-26

**Authors:** J. V. Stojanov

**Affiliations:** 1 Forensic Psychiatry, Special Hospital for Psychiatric Disorders “GornjaToponica”, Serbia; Medical faculty Nis, University of Nis, Serbia;, Nis, Serbia

## Abstract

**Introduction:**

Postnatal anxiety (PNA) in recent years, has become increasingly recognized as an important issue, as it affects a substantial number of women. Data from non-perinatal populations indicate that insomnia has a bidirectional association with anxiety.

**Objectives:**

Here we aimed to explore the association between insomnia in third trimester and PNA.

**Methods:**

We analyzed data from the hospital’s birth records and questionnaire responses from pregnancy week 32 and postnatal week 4 (n=225). Postnatal anxiety symptoms were measured using the Beck Anxiety Inventory (BAI). Anxiety disorder measurements were based on questions from the Mini-International Neuropsychiatric Interview. Insomnia was measured using the Insomnia Severity Index.

**Results:**

Among postnatal women, 8.7 % reported symptoms of at least one anxiety disorder. The observed prevalence of obsessive-compulsive disorder after delivery was 3.7%, and for social anxiety disorder 2%. Multiple regression analysis, with adjustment for several psychosocial and reproductive variables, indicated that insomnia in third trimester was significantly associated with postpartum anxiety symptoms.

**Conclusions:**

Our results suggest that anxiety disorders are prevalent in postnatal period. Healthcare professionals should be aware that women with insomnia in third trimester may have an increased risk of postnatal anxiety disorders.

**Disclosure of Interest:**

None Declared

